# Methylation-driven model for analysis of dinucleotide evolution in genomes

**DOI:** 10.1186/s12976-020-00122-x

**Published:** 2020-04-08

**Authors:** Jian-Hong Sun, Shi-Meng Ai, Shu-Qun Liu

**Affiliations:** 1 State Key Laboratory for Conservation and Utilization of Bio-Resources in Yunnan & School of Life Sciences, Yunnan University, Kunming, 650091 China; 2 College of Engineering, Honghe University, Mengzi, 661100 China; 3 Department of Applied Mathematics, Yunnan Agricultural University, Kunming, 650201 China

**Keywords:** Dinucleotide, Methylation-induced mutation, Genome composition, Genome evolution

## Abstract

**Background:**

CpGs, the major methylation sites in vertebrate genomes, exhibit a high mutation rate from the methylated form of CpG to TpG/CpA and, therefore, influence the evolution of genome composition. However, the quantitative effects of CpG to TpG/CpA mutations on the evolution of genome composition in terms of the dinucleotide frequencies/proportions remain poorly understood.

**Results:**

Based on the neutral theory of molecular evolution, we propose a methylation-driven model (MDM) that allows predicting the changes in frequencies/proportions of the 16 dinucleotides and in the GC content of a genome given the known number of CpG to TpG/CpA mutations. The application of MDM to the 10 published vertebrate genomes shows that, for most of the 16 dinucleotides and the GC content, a good consistency is achieved between the predicted and observed trends of changes in the frequencies and content relative to the assumed initial values, and that the model performs better on the mammalian genomes than it does on the lower-vertebrate genomes. The model’s performance depends on the genome composition characteristics, the assumed initial state of the genome, and the estimated parameters, one or more of which are responsible for the different application effects on the mammalian and lower-vertebrate genomes and for the large deviations of the predicted frequencies of a few dinucleotides from their observed frequencies.

**Conclusions:**

Despite certain limitations of the current model, the successful application to the higher-vertebrate (mammalian) genomes witnesses its potential for facilitating studies aimed at understanding the role of methylation in driving the evolution of genome dinucleotide composition.

## Background

The k-mer abundance analysis is widely used in genomics research [1–10]. The term k-mer refers to all possible substrings (in the 5′–3′ direction) of length k in a DNA sequence and, therefore, the k-mer frequency is a good variable for characterizing the composition of a genome’s DNA sequence. Generally, analysis of the 2-mer (i.e., dinucleotide) frequency will provide more abundant information on genome composition than a simple statistic of the 1-mer (i.e., single nucleotides A, C, G, and T) frequency.

It has been shown that in the vertebrate genomes, the CpG dinucleotide is present at a lower frequency than expected [11–14]. The reason for this is thought to be due to a high C-to-T mutation rate at the methylated CpG sites [14–18]. 5-Methylcytosine (5mc), a cytosine modified by addition of a methyl group on the fifth position of the cytidine ring, can spontaneously deaminate to form thymine (i.e., 5mc to T mutation). Unlike the cytosine (C) to uracil (U) mutation arising from the spontaneous deamination of cytosine, the 5mc to T mutation is rarely recognized and removed by DNA repair enzymes [13]. Therefore, the high level of methylation at the CpG sites explains why the mutation rate of C to T is 10–50 times higher than that of C to other nucleotides [11, 14].

Although several statistical analyses have revealed a negative correlation between the CpG and TpG/CpA levels [19–21], to the best of our knowledge, a rigorous method to predict the effects of CpG dinucleotide depletion on the changes in frequencies of all 16 dinucleotides (i.e., ApA, ApC, ApG, ApT, CpA, CpC, CpG, CpT, GpA, GpC, GpG, GpT, TpA, TpC, TpG, and TpT) and in the GC content (GC%) of vertebrate genomes is still lacking. Inspired by the substitution models [22, 23] commonly used in phylogenetic analyses and based on the neutral theory of molecular evolution, in this paper we propose a mathematical model, called the methylation-driven model (MDM), to investigate the effects of the methylation-induced CpG decay on the evolution of the genome dinucleotide composition and GC content.

## Results

For the 10 vertebrate genomes, the statistical results of the frequencies/proportions (%) of the 16 dinucleotides and the GC content are listed in Table 1, the corresponding expected values obtained by application of MDM to the initial genome state with 50% GC content and 6.25% proportion of each dinucleotide are listed in Table 2, and the application results for the other two initial genome states, i.e., with 40 and 60% GC contents, are shown in Supplementary Tables 1 and 2 (Additional file 1), respectively.

**Table 1:** Observed frequencies/proportions of the 16 dinucleotides and GC contents

	ApA/TpT	ApC/GpT	ApG/CpT	ApT	CpA/TpG	CpC/GpG	CpG	GpA/TpC	GpC	TpA	GC%
Proportion_obs_ vs. Proportion_ini_	↑	↓	↑**↓**	↑	↑	↓	↓	**↑↓**	↓	**↓↑**	↓
*Bos taurus* (cattle)	18.66%	10.20%	14.31%	7.47%	14.73%	10.74%	1.05%	**12.70%**	4.13%	**6.01%**	41.89%
*Canis lupus familiaris* (dog)	19.57%	9.69%	14.13%	7.75%	13.95%	10.84%	1.09%	12.35%	4.11%	**6.51%**	41.10%
*Gallus gallus* (chicken)	19.01%	10.40%	14.53%	7.14%	15.37%	9.61%	1.14%	11.89%	4.94%	**5.98%**	41.78%
*Pan troglodytes* (chimpanzee)	19.55%	10.08%	13.99%	7.71%	14.52%	10.43%	1.01%	11.87%	4.29%	**6.55%**	40.96%
*Danio rerio* (zebrafish)	22.12%	11.31%	**11.44%**	9.26%	14.64%	6.96%	1.79%	10.50%	3.92%	**8.06%**	36.62%
*Homo sapiens* (human)	19.63%	10.07%	13.99%	7.74%	14.50%	10.38%	0.98%	11.86%	4.26%	**6.59%**	40.99%
*Mus musculus* (house mouse)	18.06%	10.69%	14.74%	7.29%	14.95%	10.54%	0.85%	12.44%	4.13%	**6.31%**	41.93%
Papio anubis (olive baboon)	19.56%	10.16%	14.04%	7.62%	14.48%	10.39%	1.05%	11.94%	4.26%	**6.51%**	41.00%
*Ovis aries* (sheep)	18.65%	10.18%	14.35%	7.44%	14.70%	10.74%	1.08%	**12.68%**	4.17%	**6.01%**	41.95%
*Sus scrofa* (pig)	19.21%	10.07%	13.90%	7.46%	14.43%	11.09%	1.24%	11.90%	4.42%	**6.27%**	41.91%

Note: Only the autosomes of each genome were included in the statistical analyses; the symbols ‘↑’ and ‘↓’ represent an increase and decrease of the dinucleotide proportions observed (Proportion_obs_) in genomes relative to the assumed initial proportions (Proportion_ini_) obtained based on GC_ini_% = 50% (see Supplementary Table 3, Additional file 1), respectively; the values highlighted in bold exhibit changing trends incompatible with those predicted by MDM (see Table 2)

**Table 2:** Expected/calculated proportions/frequencies of the 16 dinucleotides and GC contents obtained by MDM (**GC**_**ini**_ %  **= 50**%)

	ApA/TpT	ApC/GpT	ApG/CpT	ApT	CpA/TpG	CpC/GpG	CpG	GpA/TpC	GpC	TpA	GC%
Proportion_exp_ vs. Proportion_ini_	↑	↓	↑	↑	↑	↓	↓	↓	↓	↔	↓
*Bos Taurus* (cattle)	13.82%	12.12%	13.84%	7.71%	17.70%	11.16%	1.05%	11.18%	5.17%	6.25%	44.80%
*Canis lupus familiaris* (dog)	13.72%	12.22%	13.98%	7.62%	17.66%	11.02%	1.09%	11.28%	5.15%	6.25%	44.84%
*Gallus gallus* (chicken)	13.60%	11.87%	13.66%	7.99%	17.62%	11.34%	1.14%	11.40%	5.14%	6.25%	44.89%
*Pan troglodytes* (chimpanzee)	13.74%	12.29%	13.90%	7.66%	17.74%	11.10%	1.01%	11.26%	5.05%	6.25%	44.76%
*Danio rerio* (zebrafish)	13.62%	12.34%	13.44%	7.54%	16.96%	11.56%	1.79%	11.38%	5.12%	6.25%	45.54%
*Homo sapiens* (human)	13.67%	12.44%	13.98%	7.59%	17.76%	11.02%	0.98%	11.33%	4.97%	6.25%	44.98%
*Mus musculus* (house mouse)	13.84%	12.06%	13.90%	7.79%	17.90%	11.10%	0.85%	11.16%	5.14%	6.25%	44.60%
*Papio anubis* (olive baboon)	13.66%	12.42%	13.98%	7.58%	17.70%	11.02%	1.05%	11.34%	5.01%	6.25%	44.80%
*Ovis aries* (sheep)	13.78%	12.17%	13.84%	7.69%	17.68%	11.16%	1.08%	11.22%	5.14%	6.25%	44.83%
*Sus scrofa* (pig)	13.72%	12.22%	13.96%	7.63%	17.66%	11.04%	1.09%	11.28%	5.15%	6.25%	44.99%

Note: The values presented were obtained by application of MDM to the assumed initial genome state with GC_ini_% = 50%; the symbols‘↑’, ‘↓’ and ‘↔‘represent an increase, decrease, and no-change of the expected/calculated dinucleotide proportions (Proportion_exp_) relative to the assumed initial proportions (Proportion_ini_; see Supplementary Table 3, Additional file 1), respectively

Comparison between the expected and observed trends of frequency/proportion changes relative to the initial proportions reveals that, when the initial genomes have a GC content of 50% and proportion for each dinucleotide of 6.25% (see Supplementary Table 3, Additional file 1), most of the 16 dinucleotides in most studied genomes (with the exception of TpA in all 10 genomes, GpA/TpC in cattle and sheep genomes, and ApG/CpT in zebrafish genome; see Tables 1 and 2) have a good consistency between the expected and observed changing trends. This indicates that, on the one hand, 50% GC content could be a rational assumption for the initial state of vertebrate genomes, and on the other hand, our model can achieve a good performance in predicting the changing trends in frequencies of most dinucleotides caused by the methylation-induced CpG to TpG/CpA mutations. It should be noted that, for the dinucleotide TpA, although its proportion/frequency should not be affected by cytidine methylation, the observed frequency in the eight mammalian genomes is either slightly higher or lower than the assumed initial proportion of 6.25% (ranging between 6.01 and 6.59%; see Table 1). Interestingly, in the zebrafish genome, the observed TpA frequency (8.06%) is significantly higher than the assumed initial proportion (6.25%), and in the chicken genome, the observed frequency (5.98%) is lower than those of the eight mammalian genomes.

For each of the 10 tested genomes, the comparison between the observed and expected (obtained with the initial GC content of 50%) frequencies/proportions of the 16 dinucleotides shows an acceptable conformance for most of the 16 dinucleotides (Supplementary Fig. 1, Additional file 1). In order to check whether our model could replicate the observed frequencies/proportions of the 16 dinucleotides, for each of the 10 vertebrate genomes, we have performed the paired t-test of the null hypothesis regarding the differences between the predicted (Table 2) and observed frequencies (Table 1) of the 16 dinucleotides. The results (see Supplementary Fig. 1, Additional file 1) show that all the 10 *P*-values are close to 1 (> 0.05), indicating that the null hypothesis cannot be rejected at the 95% confidence level and, hence, the differences between the predicted and observed frequencies are statistically acceptable for each tested genome.

**Fig. 1:**
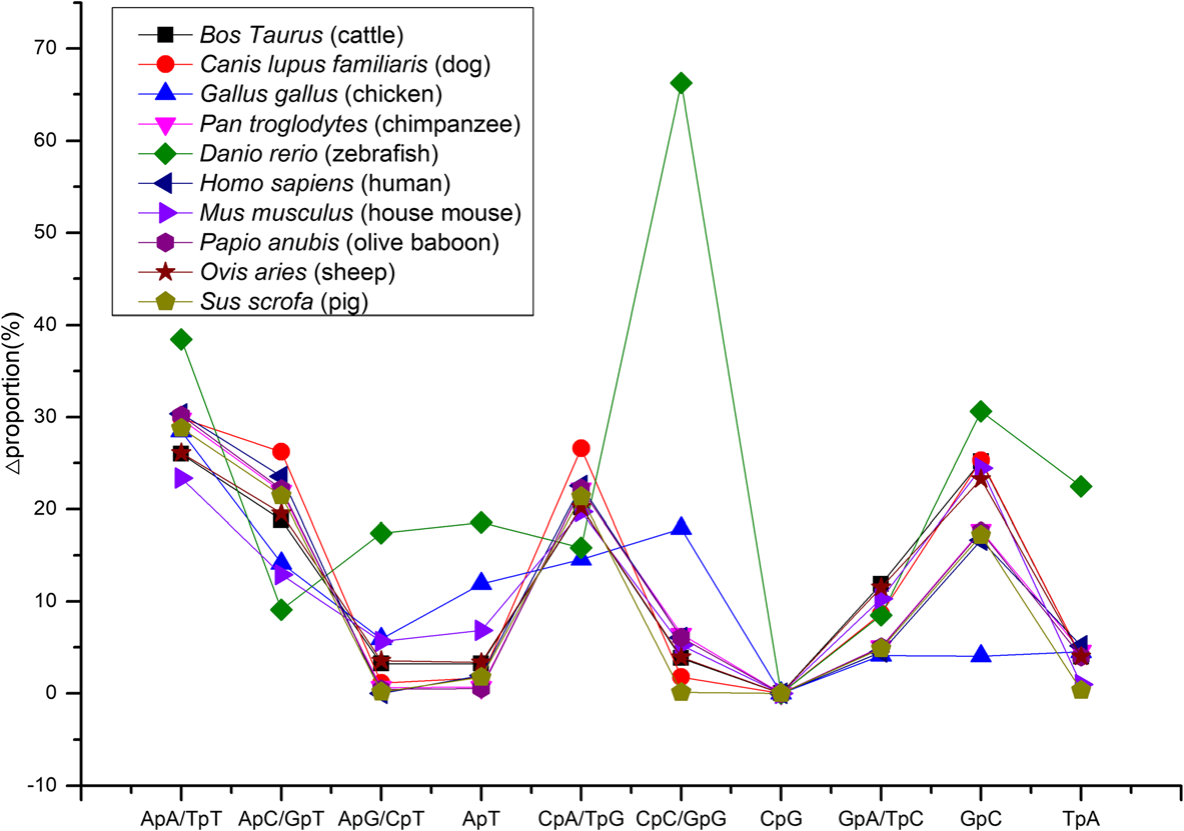
Relative differences between the expected and observed proportions of the 16 dinucleotides

In order to evaluate the relative difference between the expected (Proportion_exp_) and observed (Proportion_obs_) dinucleotide frequencies/proportions, the value of ΔProportion, defined as the ratio of |(Proportion_exp_ - Proportion_obs_)| to Proportion_obs_, was calculated. Figure 1 shows the ΔProportion values of all 16 nucleotides (calculated using Proportion_exp_ obtained from the assumed initial genome state with GC content of 50%) for the 10 vertebrate genomes investigated. For the eight mammalian genomes, most of the 16 dinucleotides have the ΔProportion values either lower than 10% (ApG/CpT, ApT, CpC/GpG, CpG, GpA/TpC, and TpA) or clustering around 20% (ApC/GpT, CpA/TpG, and GpC), indicating a high similarity between the expected and observed frequencies/proportions of them; only two dinucleotides (i.e., ApA/TpT) in few mammal genomes have the ΔProportion values greater than 30%, which indicate relatively large deviations of the expected frequencies from the observed ones. In contrast, the results for the lower-vertebrate (zebrafish) genome do not seem to be satisfactory because several dinucleotides (e.g., CpC/GpG and ApA/TpT) have the expected proportions/frequencies that radically deviate from the observed ones (**Δ**Proportion > 35%). Also worth noting is that the ΔProportion values of some dinucleotides in the lower-vertebrate genomes (e.g., ApA/TpT, ApG/CpT, ApT, CpC/GpG, and TpA in the zebrafish genome; ApT, CpC/GpG, and GpC in the chicken genome) do not cluster around those of the mammal genomes. Overall, the above results indicate that, despite the different application effects on the higher-vertebrate (mammalian) and lower-vertebrate genomes, our model displays a better performance when applied to the mammalian genomes.

**Fig. 2:**
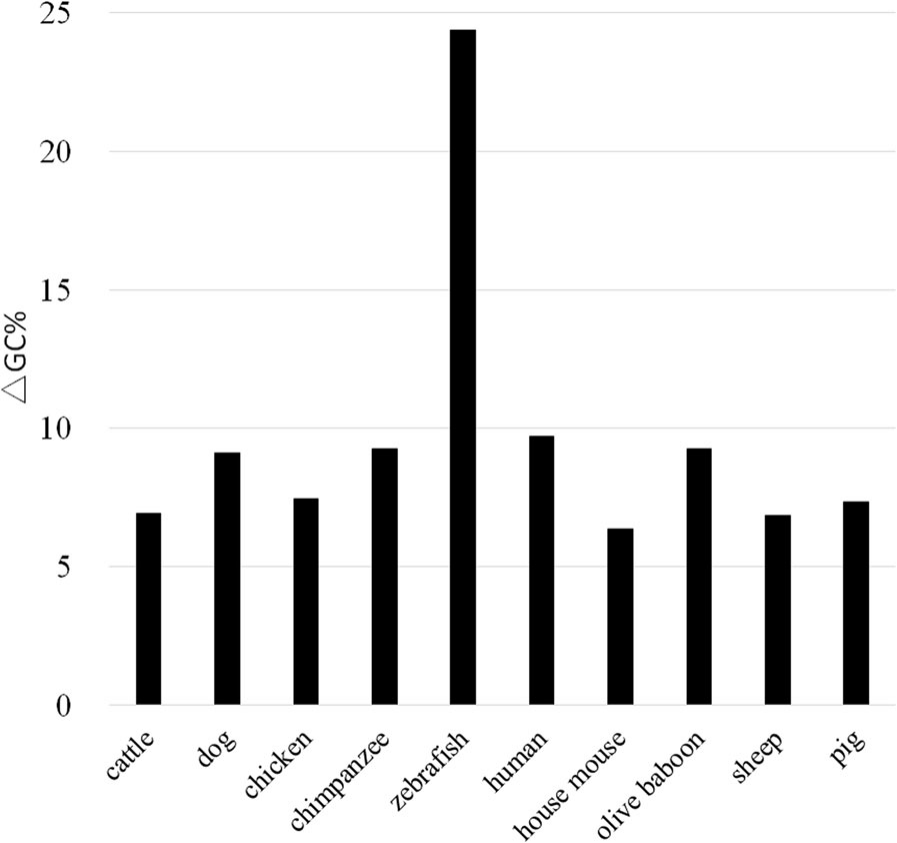
Relative differences between the expected and observed GC contents

Changes in the GC content due to cytidine methylation are only related to the number of CpG to TpG/CpA mutations. The relative difference between the expected (GC%_exp_) and observed (GC%_obs_) GC contents was evaluated by calculating the ΔGC% value: ΔGC% = |(GC%_exp_ - GC%_obs_)| / GC%_obs_. Figure 2 shows ΔGC% values (calculated using GC%_exp_ obtained from the assumed initial state of a genome with 50% GC content) for the 10 genomes, out of which nine have ΔGC% values less than 10%, indicating a high agreement between the calculated and observed GC contents. The only exception is the zebrafish genome, for which the high ΔGC% value (24.4%) indicates a large deviation of the predicted GC content from the observed one.

## Discussion

In this paper, we have proposed a mathematical model based on the neutral theory of molecular evolution to analyze the effects of the methylation-induced CpG to TpG/CpA mutations on the evolution of the genome dinucleotide composition and GC content**.** The model hypothesizes that the neutral mutations (i.e., non-CpG-to-TpG/CpA mutations) would have no effect on the evolution of genome composition. What needs to be highlighted is that the model established here mainly focuses on the methylation-driven evolution of the dinucleotide composition. The previously proposed substitution models, such as the JC69 [22], K80 [23], and TN93 [2] models, despite being widely used in molecular phylogenetic analyses and genome evolution studies, use the rates of the single-nucleotide (1-mer) substitutions as the main parameters. Moreover, these models cannot answer the question of why the observed CpG frequency in the vertebrate genomes is much lower than that expected from the GC content. Although the high rate of the methylation-induced CpG to TpG/CpA mutations can explain the globally reduced frequency of the CpG dinucleotide compared with its expected frequency, there has been a lack of theoretical models with which to predict the mutation effect on the genome composition in terms of 2-mers. Therefore, we propose the MDM through which the methylation-induced changes in frequencies/proportions of the 16 dinucleotides and the GC content can be predicted based on an assumed initial state of a genome.

When modeling the genome evolution, it is inevitable to make assumptions regarding some parameters that cannot be directly obtained. As a matter of fact, the effectiveness of our model largely depends on the validity of the assumed parameters, which in turn depends on the composition characteristics of genomes of interest. For example, for a majority of the 16 dinucleotides and the GC content, their expected/calculated frequencies/content in the zebrafish genome are distinctly different from those in the mammalian genomes (Figs. 1 and 2), and this could be attributed to the large difference in the genome composition between zebrafish and mammals [24]. Specifically, the assumed initial state of the genome can have a large influence on the performance of our model. For example, when the dinucleotide proportions in the initial genome state were assigned based on the assumed GC contents of 40 and 60%, the expected/predicted values for more than half of the 16 dinucleotides and GC contents radically deviate from the observed ones in all the 10 tested genomes (Table 1 and Supplementary Tables 1 and 2, Additional file 1). Even in the case of the “reasonable” initial genome state with the assumed GC content of 50%, the expected proportions/frequencies for ApA/TpT differ from the observed ones by more than 25% in most of the 10 genomes (Fig. 1). We consider that it is the assumed initial proportions of ApA/TpT in the genome that leads to the large deviations from the observed values, while the model itself does not account for these.

It should be noted that there are still shortcomings in MDM regarding its application. Since the model is built based on the neutral mutation theory of molecular evolution, it assumes that the numbers of substitutions between any two single nucleotides are equal (e.g., the numbers of mutations from C to A and from A to C are the same) and, hence, all mutations except for CpG to TpG/CpA will have no effect on the evolution of the genome dinucleotide composition. In fact, mutation bias is prevalent in nature. In Kimura’s two-parameter model [23], rates of transition (α) and transversion (β) substitutions are different and, furthermore, there is no evidence indicating that the CpG to TpG/CpA mutation is the sole factor influencing the rates of transitions and transversions. Ignoring the effects of non-CpG-to-TpG/CpA mutations on the evolution of the genome dinucleotide composition can be expected to produce deviations from the observed values. Moreover, there are limitations in the parameter estimation methods of the presented model, which assume that the rates of the methylation-induced CpG to TpG/CpA mutations are independent of the sequence context of CpG. In fact, the CpG to TpG/CpA mutations are non-neutral [25] and their substitution rates are sequence context dependent [26, 27].

## Conclusions

In this work, we have proposed a mathematical model to investigate the effects of the methylation-induced CpG to TpG/CpA mutations on the evolution of genome composition in terms of the 2-mers and GC content. The application of our model to the 10 vertebrate genomes has achieved a good consistency between the predicted and observed trends of changes in the GC content and frequencies of most of the 16 dinucleotides with respect to their assumed initial values; moreover, for the 10 tested genomes, quantitative evaluations of the relative differences in the dinucleotide frequency and GC content between the expected and observed values show a better performance of our model when applied to the mammalian genomes than to the lower vertebrate genomes. Despite the capability of MDM to quantify the effects of the methylation-induced CpG decay on the evolution of the genome dinucleotide composition and GC content, there are still limitations to the current model because i) the rates of the methylation-induced CpG to TpG/CpA mutations are dependent (rather than independent, as assumed in the current model) on the sequence context of CpG sites and, ii) the proportions of the 16 dinucleotides in the initial state of different vertebrate genomes may not be simply identical but depend on the genome composition characteristics. As a result, future efforts in improving the model should be directed toward i) improving the parameter estimation method to make the estimated parameters reflect the context-dependent rates of methylation-induced CpG to TpG/CpA mutations and, ii) realizing the customization of the initial dinucleotide proportions. These two points could be addressed by assigning appropriate weighting coefficients to the parameters as estimated by the Trinucleotide-method and using the genome-composition-based bias factors to calibrate the proportions of the 16 dinucleotides in the initial state of a genome of interest, respectively.

## Methods

According to the neutral mutation theory of molecular evolution [28], most evolutionary changes and most of the variability within species at the molecular level are not caused by natural selection, but by random genetic drift of selectively neutral mutations. Since neutral mutations are those that do not affect the survival or reproduction of an organism, they are expected to have a minor effect on the genome dinucleotide composition in the long-term evolutionary process. However, in vertebrate genomes, CpG hypermutability caused by the cytosine methylation clearly exceeds the random expectation. In vertebrate genomes, the observed frequency of CpG dinucleotides is much lower than that expected based on the GC content, implying that the methylation-induced CpG to TpG/CpA mutations exert a substantial effect on the evolution of the genome dinucleotide composition.

MDM proposed in this study focuses on the effects of the methylation-induced CpG to TpG/CpA mutations on the changes in frequencies/proportions of all 16 dinucleotides and in the GC content. The basic hypothesis of MDM is that the cytidine methylation is a key factor that causes the different rates of the transition (α) and transversion (β) substitutions, which under Kimura’s model [23] are considered to drive genome evolution.

### Model construction

We assume that in a DNA sequence, each CpG to TpG/CpA substitution is independent from its context. In the case of NpCpG (N = A, C, G, or T), the outcomes of the methylation-induced CpG to TpG mutation on the dinucleotide components can be dissected as follows: the number of CpG is reduced by 1, the number of TpG is increased by 1, and the changes in the numbers of other dinucleotides depend on the nucleotide type of N; for example, if N is A, the number of the dinucleotide ApC will be reduced by 1, while that of ApT will be increased by 1. Let *P*_*A*_, *P*_*C*_, *P*_*G*_, and *P*_*T*_ represent the probabilities of N beating A, C, G and T, respectively, then *P*_*A*_ + *P*_*C*_ + *P*_*G*_ + *P*_*T*_ = 1. The changes in the numbers of all 16 dinucleotides in the context of NpCpG upon mutation can be described by the matrix D:

1 $$ \mathrm{D}=\left({d}_{ij}\right)=\left(\begin{array}{cccc}{d}_{AA}& {d}_{AC}& {d}_{AG}& {d}_{AT}\\ {}{d}_{CA}& {d}_{CC}& {d}_{CG}& {d}_{CT}\\ {}{d}_{GA}& {d}_{GC}& {d}_{GG}& {d}_{GT}\\ {}{d}_{TA}& {d}_{TC}& {d}_{TG}& {d}_{TT}\end{array}\right)=\left(\begin{array}{cccc}0& -{P}_A& 0& {P}_A\\ {}0& -{P}_C& -1& {P}_C\\ {}0& -{P}_G& 0& {P}_G\\ {}0& -{P}_T& 1& {P}_T\end{array}\right) $$

where *d*_*ij*_ represents the change in the probability of a dinucleotide composed of nucleotides *i* and *j* upon NpCpG to NpTpG mutation (*i, j* = A, C, G, or T), and a negative element, e.g., *d*_*AC*_ = −*P*_*A*_, indicates that the number of the corresponding dinucleotide ApC is reduced, and vice versa. Similarly, changes in the numbers of 16 dinucleotides upon CpGpM to CpApM (M = A, C, G, or T) mutation can be represented by the matrix D*’*; where *P’*_*A*_, *P’*_*C*_, *P’*_*G*_, and *P’*_*T*_ denote the probabilities of M beating A, C, G, and T, respectively, and *P’*_*A*_ + *P’*_*C*_ + *P’*_*G*_ + *P’*_*T*_ = 1.

2 $$ {\mathrm{D}}^{\prime }=\left({d}_{ij}^{\prime}\right)=\left(\begin{array}{cccc}{d}_{AA}^{\prime }& {d}_{AC}^{\prime }& {d}_{AG}^{\prime }& {d}_{AT}^{\prime}\\ {}{d}_{CA}^{\prime }& {d}_{CC}^{\prime }& {d}_{CG}^{\prime }& {d}_{CT}^{\prime}\\ {}{d}_{GA}^{\prime }& {d}_{GC}^{\prime }& {d}_{GG}^{\prime }& {d}_{GT}^{\prime}\\ {}{d}_{TA}^{\prime }& {d}_{TC}^{\prime }& {d}_{TG}^{\prime }& {d}_{TT}^{\prime}\end{array}\right)=\left(\begin{array}{cccc}P{\prime}_A& P{\prime}_C& P{\prime}_G& P{\prime}_T\\ {}1& 0& -1& 0\\ {}-P{\prime}_A& -P{\prime}_C& -P{\prime}_G& P{\prime}_T\\ {}0& 0& 0& 0\end{array}\right) $$

The principle of complementary base pairing dictates that the number of NpCpG to NpTpG mutations on the forward strand is the same as that of CpGpM to CpApM mutations on the reverse strand; furthermore, this principle dictates that the CpG to TpG mutations caused by methylation on the reverse strand corresponds to the CpG to CpA mutations on the forward strand, and vice versa. As a result, both the CpG to TpG and CpG to CpA mutations observed on any one of the two strands are the consequence of cytidine methylation of the CpG dinucleotides, although the summation of their numbers doubles the number of the actually depleted CpG dinucleotides. If the number of CpG to TpG/CpA mutations is set to H, the changes in the numbers of the 16 dinucleotides can be calculated by the matrix Q:

3 $$ \mathrm{Q}=\frac{\mathrm{H}}{2}\left(\mathrm{D}+{\mathrm{D}}^{\prime}\right)=\frac{\mathrm{H}}{2}\left(\begin{array}{cccc}{P}_A^{\prime }& {P}_C^{\prime }-{P}_A& {P}_G^{\prime }& {P}_T^{\prime }+{P}_A\\ {}1& -{P}_C& -2& {P}_C\\ {}-{P}_A^{\prime }& -{P}_C^{\prime }-{P}_G& -{P}_G^{\prime }& {P}_G-{P}_T^{\prime}\\ {}0& -{P}_T& 1& {P}_T\end{array}\right) $$

The values of the eight parameters *P*_*A*_, *P*_*C*_, *P*_*G*_, *P*_*T*_, *P’*_*A*_, *P’*_*C*_, *P’*_*G*_, and *P’*_*T*_ vary between 0 and 1. Before estimating the parameters of the model, we cannot determine the positive or negative values of $\Big({P}_C^{\prime }-{P}_A$) and (${P}_G-{P}_T^{\prime }$). However, it is clear from the matrix Q that for 14 of the 16 dinucleotides (with the exception of ApC (*P’*_*C*_ - *P*_*A*_) and GpT (*P*_*G*_ - *P’*_*T*_)), their changes in number can be directly inferred without requiring parameter estimation. Table 3 lists the changing trends in the numbers of the 14 dinucleotides upon CpG to TpG/CpA mutations inferred directly from the matrix Q.

**Table 3:** CpG to TpG/CpA mutation-caused changing trends in the numbers of dinucleotides

	ApA/TpT	ApC/GpT	ApG/CpT	ApT	CpA/TpG	CpC/GpG	CpG	GpA/TpC	GpC	TpA
Changing trend	↑	undetermined	↑	↑	↑	↓	↓	↓	↓	↔

Note: The changing trends are inferred directly from the matrix Q, with the symbols‘↑’, ‘↓’ and ‘↔’ representing an increase, decrease, and no-change, respectively, in the numbers of corresponding dinucleotides; ‘undetermined’ denotes that the changing trends in the numbers of ApC/GpT cannot be determined from the matrix Q without parameter estimation

### Parameter estimation

In the matrix Q, the values for the parameters *P*_*A*_, *P*_*C*_, *P*_*G*_, *P*_*T*_, *P’*_*A*_, *P’*_*C*_, *P’*_*G*_, and *P’*_*T*_ are unknown. To determine the change in the number of each dinucleotide as a function of H (i.e., the number of CpG to TpG/CpA mutations), each parameter value should be estimated. Based on the assumption that the rate of the methylation-induced CpG-to-TpG/CpA mutations is independent of the sequence context of CpG sites, we propose two methods for parameter estimation.

Trinucleotide-method: Using a simple ratio approach, the parameters *P*_*A*_, *P*_*C*_, *P*_*G*_, and *P*_*T*_ can be calculated as the proportion of ApCpG, CpCpG, GpCpG, and TpCpG among all NpCpG trinucleotides, respectively. Accordingly, *P’*_*A*_, *P’*_*C*_, *P’*_*G*_, and *P’*_*T*_ can be obtained through calculating the proportion of CpGpA, CpGpC, CpGpG, and CpGpT among all CpGpM trinucleotides, respectively. Since most CpG islands remain unmethylated in normal cells [29, 30], they are excluded from parameter estimation. In addition, the CpG sites located within the coding regions could also be excluded due to the high selection pressure on these regions. Nevertheless, since the proportion of CpGs in the coding regions out of the total CpGs is small, we expect that the inclusion of the coding-region CpGs would have a negligible effect on the estimation results. Through counting all the eight trinucleotides in the context of NpCpG and CpGpM, the parameters can be calculated by eqs. (4) and (5):

4 $$ \left.\begin{array}{c}{P}_A=\frac{S_{\mathrm{ACG}}}{S_{\mathrm{ACG}}+{S}_{\mathrm{TCG}}+{S}_{\mathrm{CCG}}+{S}_{\mathrm{GCG}}}\\ {}{P}_C=\frac{S_{\mathrm{CCG}}}{S_{\mathrm{ACG}}+{S}_{\mathrm{TCG}}+{S}_{\mathrm{CCG}}+{S}_{\mathrm{GCG}}}\\ {}{P}_G=\frac{S_{\mathrm{GCG}}}{S_{\mathrm{ACG}}+{S}_{\mathrm{TCG}}+{S}_{\mathrm{CCG}}+{S}_{\mathrm{GCG}}}\\ {}{P}_T=\frac{S_{\mathrm{TCG}}}{S_{\mathrm{ACG}}+{S}_{\mathrm{TCG}}+{S}_{\mathrm{CCG}}+{S}_{\mathrm{GCG}}}\end{array}\right\} $$

5 $$ \left.\begin{array}{c}P{\prime}_A=\frac{S_{\mathrm{CGA}}}{S_{\mathrm{CGA}}+{S}_{\mathrm{CGT}}+{S}_{\mathrm{CGC}}+{S}_{\mathrm{CGG}}}\\ {}P{\prime}_C=\frac{S_{\mathrm{CGC}}}{S_{\mathrm{CGA}}+{S}_{\mathrm{CGT}}+{S}_{\mathrm{CGC}}+{S}_{\mathrm{CGG}}}\\ {}P{\prime}_G=\frac{S_{\mathrm{CGG}}}{S_{\mathrm{CGA}}+{S}_{\mathrm{CGT}}+{S}_{\mathrm{CGC}}+{S}_{\mathrm{CGG}}}\\ {}P{\prime}_T=\frac{S_{\mathrm{CGT}}}{S_{\mathrm{CGA}}+{S}_{\mathrm{CGT}}+{S}_{\mathrm{CGC}}+{S}_{\mathrm{CGG}}}\end{array}\right\} $$

where S_ACG_, S_CCG_, S_GCG_, and S_TCG_ are the numbers of ApCpG, CpCpG, GpCpG, and TpCpG, respectively, and S_CGA_, S_CGC_, S_CGG_, and S_CGT_ are the numbers of CpGpA, CpGpC, CpGpG, and CpGpT, respectively, in a genome sequence.

GC-method: Because of the sequence complementarity between the forward and reverse strands of DNA [31], the parameters have the following relationships: *P*_*A*_ ≈ *P’*_*T*_, *P*_*C*_ ≈ *P’*_*G*_, *P*_*G*_ ≈ *P’*_*C*_, and *P*_*T*_ ≈ *P’*_*A*_. Therefore, the eight parameters can be estimated based on the GC content in a genome. If the GC content is *p*, then the AT content is (1 – *p*), and the following relations can be obtained:

6 $$ \left.\begin{array}{c}{P}_A=\frac{1-\mathrm{p}}{2}\\ {}{P}_C=\frac{\mathrm{p}}{2}\ \\ {}{P}_G=\frac{\mathrm{p}}{2}\ \\ {}{P}_T=\frac{1-\mathrm{p}}{2}\end{array}\right\} $$

7 $$ \left.\begin{array}{c}P{\prime}_A\approx \frac{1-\mathrm{p}}{2}\\ {}P{\prime}_C\approx \frac{\mathrm{p}}{2}\ \\ {}P{\prime}_G\approx \frac{\mathrm{p}}{2}\ \\ {}P{\prime}_T\approx \frac{1-\mathrm{p}}{2}\end{array}\right\} $$

For example, if the GC content is 40%, then *P*_*A*_ = *P*_*T*_ = *P’*_*T*_ = *P’*_*A*_ = 0.3 and *P*_*C*_ = *P*_*G*_ = *P’*_*C*_ = *P’*_*G*_ = 0.2.

It should be noted that in the practical application of MDM, the trinucleotide-method should be preferred over the GC-method. In the application example below, the parameters are estimated based on the current state of a genome and are assumed to be constant during the genome evolution. In fact, the cumulative mutations of CpG to TpG/CpA will inevitably lead to a reduction in the GC content. Therefore, if the GC-method is used, the four estimated parameters (i.e., *P*_*C*_, *P*_*G*_, *P’*_*C*_, and *P’*_*G*_) will be prone to errors due to the large changes in the GC content between the assumed initial state and current state of a genome.

### Application example

The constructed MDM was applied to predict the effects of CpG to TpG/CpA mutations on the evolution of the dinucleotide composition in vertebrate genomes starting from three assumed initial states. The source code used is publicly available at https://github.com/sparkhonghe/MDM.

Ten vertebrate genomes, of which eight are from mammals (higher vertebrates) and two from non-mammals (lower vertebrates, i.e., *Gallus gallus* (chicken) and *Danio rerio* (zebrafish)) (see Supplementary Table 4, Additional file 1), were obtained from the genome database of the National Center for Biotechnology Information (NCBI; https://www.ncbi.nlm.nih.gov). To avoid artifacts arising from sex-specific effects, only the sequence from autosomes was included in the statistics of the frequencies/proportions (%) of the 16 dinucleotides and the GC content in these 10 genomes.

There are three basic assumptions in application of MDM: i) the loss of CpG dinucleotides in a vertebrate genome is exclusively caused by cytidine methylation; ii) the rate of the methylation-induced CpG to TpG/CpA mutations is independent of the sequence context of CpG sites; and iii) the total number of dinucleotides in the genome remains constant during evolution. In addition, the model’s application also needs to make assumptions about the proportions/frequencies of the 16 dinucleotides in the initial state of a genome, which can be obtained based on the assumed initial GC content (GC_ini_%). In this study, three assumed initial genome states, i.e., in which the proportions of the 16 dinucleotides were obtained (see eq. (8) below) according to the GC_ini_% of 40% (see Supplementary Table 5, Additional file 1), 50% (Supplementary Table 3, Additional file 1), and 60% (Supplementary Table 6, Additional file 1), respectively, were tested. It should be noted that, under the same GC content, all the 10 genomes possess the same initial state in terms of the frequency/proportion of each dinucleotide.

Given the GC content, the frequencies/proportions of the 16 dinucleotides (NpM_ini_%) in the assumed initial state of a genome can be calculated using the following equation:

8 $$ {\mathrm{N}\mathrm{pM}}_{\mathrm{ini}}\%=\left({\mathrm{N}}_{\mathrm{num}}\ast {\mathrm{M}}_{\mathrm{num}}\right)/{\mathrm{L}}^2 $$

where M and N = A, C, G, or T, N_num_ and M_num_ represent the numbers of nucleotides N and M in the initial genome, respectively, and L is the genome length. As a result, N_num_/L and M_num_/L designate the frequencies of N and M, respectively. For example, assuming that the GC content is 50% and genome length is 100, then in the initial state of this genome, the number of each of the four nucleotides (A, C, G and T) is 25, and the frequency of each of the 16 dinucleotides (NpM_ini_%) is 25 * 25/100^2^ = 6.25%. Note that the eq. (8) can also be used for estimating the expected frequency of CpG in the current state of a genome if we take N_num_/L and M_num_ / L as the observed frequencies of C and G (i.e., one-half of the observed GC content), respectively, in the genome.

As rationalized above, the GC content can vary during the evolution of a genome due to continuous CpG depletion. Therefore, we adopt the “Trinucleotide-method” to estimate the parameters P_A_, P_C_, P_G_, P_T_, P’_A_, P’_C_, P’_G_, and P’_T_ in the matrix Q. The total number of the depleted CpG dinucleotides (H) in a genome can be calculated using the following equations:

9 $$ \mathrm{H}=\left(\mathrm{CpG}{\%}_{\mathrm{ini}}-\mathrm{CpG}{\%}_{\mathrm{obs}}\right)\ast {\mathrm{N}}_{\mathrm{N}\mathrm{pM}} $$

10 $$ {\mathrm{N}}_{\mathrm{N}\mathrm{pM}}=\mathrm{L}-1 $$

where CpG%_ini_ and CpG%_obs_ is the assumed initial proportion (see Supplementary Tables 3, 5, and 6, Additional file 1) and the observed frequency of CpG (Table 1) in the genome, respectively, N_NpM_ is the total number of dinucleotides in the genome, and L is the genome length after removing gaps (Supplementary Table 4, Additional file 1).

The CpG island, which is defined as a stretch of DNA sequence with length > 200 bp, GC content > 50%, and observed-to-expected CpG ratio > 0.6 [13, 32], was identified using the CpG Island Searcher [33]. After removing CpG islands, NpCpG and CpGpM trinucleotides in each of the 10 vertebrate genomes were counted using an in-house Java program (for results, see Supplementary Table 7, Additional file 1), and the eight parameters were then obtained with eqs. (4) and (5). Table 4 lists the estimated parameters for all the 10 vertebrate genomes.

**Table 4:** Parameters estimated by statistics of the trinucleotides NpCpG and CpGpM

	*P*_*A*_	*P*_*C*_	*P*_*G*_	*P*_*T*_	*P’*_*A*_	*P’*_*C*_	*P’*_*G*_	*P’*_*T*_
*Bos Taurus* (cattle)	0.2804	0.2590	0.2075	0.2531	0.2525	0.2072	0.2588	0.2815
*Canis lupus familiaris* (dog)	0.2654	0.2857	0.2128	0.2361	0.2360	0.2127	0.2856	0.2657
*Gallus gallus* (chicken)	0.3397	0.2284	0.2161	0.2158	0.2157	0.2166	0.2281	0.3396
*Pan troglodytes* (chimpanzee)	0.2683	0.2679	0.2285	0.2353	0.2349	0.2286	0.2675	0.2690
*Danio rerio* (zebrafish)	0.2887	0.2092	0.2523	0.2497	0.2498	0.2527	0.2096	0.2879
*Homo sapiens* (human)	0.2537	0.2818	0.2422	0.2223	0.2218	0.2420	0.2818	0.2544
*Mus musculus* (house mouse)	0.2856	0.2606	0.2053	0.2485	0.2483	0.2052	0.2605	0.2860
*Papio anubis* (olive baboon)	0.2553	0.2837	0.2384	0.2226	0.2224	0.2386	0.2837	0.2554
*Ovis aries* (sheep)	0.2778	0.2598	0.2150	0.2474	0.2466	0.2148	0.2595	0.2790
*Sus scrofa* (pig)	0.2663	0.2838	0.2137	0.2361	0.2356	0.2135	0.2840	0.2668

Note: Only the autosomes of each genome were included in the statistical analyses

Once the eight parameters (Table 4) were obtained and the number of CpG to TpG/CpA mutations (H) was determined (eq. (9)), the changes in the numbers of the 16 dinucleotides in a genome from the assumed initial state to the current state can be derived from the matrix Q (eq. (3)). Finally, for each of the 16 dinucleotides, its predicted/expected number in the current genome state was obtained by adding the predicted number of changes to the assumed initial number, followed by converting to the proportion of the total number of all 16 dinucleotides to facilitate comparison.

The expected GC content (GC%_exp_) in a genome was obtained using the following equation:

11 $$ \mathrm{GC}{\%}_{\mathrm{exp}}={\mathrm{GC}}_{\mathrm{ini}}\%-\mathrm{H}/\mathrm{L} $$

## Supplementary information

**Additional file 1: Supplementary Table 1**. Expected/calculated proportions/frequencies of the 16 dinucleotides and GC contents obtained by MDM (GC_ini_% = 40%). **Supplementary Table 2.** Expected/calculated proportions/frequencies of the 16 dinucleotides and GC contents obtained by MDM (GC_ini_% = 60%). **Supplementary Table 3.** Proportions/frequencies of the 16 dinucleotides in the assumed initial state of genomes with GC_ini_% = 50%. **Supplementary Table 4.** Information of the 10 vertebrate genomes. **Supplementary Table 5**. Proportions/frequencies of the 16 dinucleotides in the assumed initial state of genomes with GC_ini_% = 40%. **Supplementary Table 6.** Proportions/frequencies of the 16 dinucleotides in the assumed initial state of genomes with GC_ini_% = 60%. **Supplementary Table 7**. Numbers of the trinucleotides NpCpG and CpGpM in the 10 vertebrate genomes. **Supplementary Fig. 1**. Comparison between the observed and expected frequencies/proportions of the 16 dinucleotides. Note that the expected frequencies were obtained using GC_ini_% = 50%. *P*-value shown in the inserted box was obtained by performing the paired t-test on the observed and expected frequencies of the 16 dinucleotides for each genome.

## Data Availability

All data generated or analyzed during this study are included in this published article and its supplementary information files.

## Abbreviation

MDM: Methylation-driven model
