# Supplementary material for: Methylation-driven model for analysis of dinucleotide evolution in genomes
Source: Theor Biol Med Model. 2020 Apr 8;17:3. doi: 10.1186/s12976-020-00122-x (PMC7140373; doi:10.1186/s12976-020-00122-x)
Supplement: Supplementary file 1 — Additional file 1: Supplementary Table 1. Expected/calculated proportions/frequencies of the 16 dinucleotides and GC contents obtained by MDM (GCini% = 40%). Supplementary Table 2. Expected/calculated proportions/frequencies of the 16 dinucleotides and GC contents obtained by MDM (GCini% = 60%). Supplementary Table 3. Proportions/frequencies of the 16 dinucleotides in the assumed initial state of genomes with GCini% = 50%. Supplementary Table 4. Information of the 10 vertebrate genomes. Supplementary Table 5. Proportions/frequencies of the 16 dinucleotides in the assumed initial state of genomes with GCini% = 40%. Supplementary Table 6. Proportions/frequencies of the 16 dinucleotides in the assumed initial state of genomes with GCini% = 60%. Supplementary Table 7. Numbers of the trinucleotides NpCpG and CpGpM in the 10 vertebrate genomes. Supplementary Fig. 1. Comparison between the observed and expected frequencies/proportions of the 16 dinucleotides. Note that the expected frequencies were obtained using GCini% = 50%. P-value shown in the inserted box was obtained by performing the paired t-test on the observed and expected frequencies of the 16 dinucleotides for each genome. [file 12976_2020_122_MOESM1_ESM.docx]

# Methylation-Driven Model for Analysis of Dinucleotide Evolution in Genomes

Jian-Hong Sun^1,2#^, Shi-Meng Ai^3#^, Shu-Qun Liu^1*^

^1^ State Key Laboratory for Conservation and Utilization of Bio-Resources in Yunnan & School of Life Sciences, Yunnan University, Kunming 650091, China

^2^ College of Engineering, Honghe University, Mengzi 661100, China

^3^ Department of Applied Mathematics, Yunnan Agricultural University, Kunming 650201, China

*Correspondence: [shuqunliu@ynu.edu.cn](mailto:shuqunliu@ynu.edu.cn) (SQL)

^#^These authors contributed equally to this work.

Supplementary Table 1 Expected/calculated proportions/frequencies of the 16 dinucleotides and GC contents obtained by MDM ($\mathbf{GC}_{\mathbf{ini}}\boldsymbol{\%=40\%}$)

|  | ApA/TpT | ApC/GpT | ApG/CpT | ApT | CpA/TpG | CpC/GpG | CpG | GpA/TpC | GpC | TpA | GC% |
| --- | --- | --- | --- | --- | --- | --- | --- | --- | --- | --- | --- |
| Proportion_exp_ vs. Proportion_ini_ | ↑ | ↓ | ↑ | ↑ | ↑ | ↓ | ↓ | ↓ | ↓ | ↔ | ↓ |
| Proportion_obs_ vs*.* Proportion_ini_ | ↑ | ↓ | ↑↓ | ↑↓ | ↑ | ↑↓ | ↓ | ↑↓ | ↑↓ | ↓ | ↑↓ |
| *Bos Taurus* (cattle) | 19.31% | 11.62% | 13.35% | 10.46% | 17.20% | 6.65% | -1.20% | 10.69% | 2.92% | 9.00% | 34.80% |
| *Canis lupus familiaris* (dog) | 19.22% | 11.73% | 13.47% | 10.37% | 17.16% | 6.53% | -1.16% | 10.78% | 2.90% | 9.00% | 34.84% |
| *Gallus gallus* (chicken) | 19.10% | 11.37% | 13.17% | 10.74% | 17.11% | 6.83% | -1.11% | 10.90% | 2.89% | 9.00% | 34.89% |
| *Pan troglodytes* (chimpanzee) | 19.23% | 11.79% | 13.40% | 10.41% | 17.24% | 6.60% | -1.24% | 10.77% | 2.80% | 9.00% | 34.76% |
| *Danio rerio* (zebrafish) | 19.11% | 11.84% | 12.93% | 10.29% | 16.46% | 7.07% | -0.46% | 10.89% | 2.87% | 9.00% | 35.54% |
| *Homo sapiens* (human) | 19.17% | 11.94% | 13.49% | 10.34% | 17.27% | 6.51% | -1.27% | 10.83% | 2.72% | 9.00% | 34.98% |
| *Mus musculus* (house mouse) | 19.34% | 11.57% | 13.41% | 10.54% | 17.40% | 6.59% | -1.40% | 10.66% | 2.89% | 9.00% | 34.60% |
| *Papio anubis* (olive baboon) | 19.16% | 11.91% | 13.48% | 10.33% | 17.20% | 6.52% | -1.20% | 10.84% | 2.76% | 9.00% | 34.80% |
| *Ovis aries* (sheep) | 19.28% | 11.67% | 13.34% | 10.44% | 17.17% | 6.66% | -1.17% | 10.72% | 2.89% | 9.00% | 34.83% |
| *Sus scrofa* (pig) | 19.18% | 11.73% | 13.42% | 10.34% | 17.01% | 6.58% | -1.01% | 10.82% | 2.93% | 9.00% | 34.99% |

*Note: The values presented were obtained by application of MDM to the assumed initial genome state with GC_ini_% = 40%; the symbols‘↑’, ‘↓’ and ‘*↔*’represent an increase, decrease, and no-change of the dinucleotide proportions expected/calculated (Proportion_exp_) or observed (Proportion_obs_；see Table 1) in genomes relative to the assumed initial proportions (Proportion_ini_; see Supplementary Table 5), respectively.*

Supplementary Table 2 Expected/calculated proportions/frequencies of the 16 dinucleotides and GC contents obtained by MDM ($\mathbf{GC}_{\mathbf{ini}}\boldsymbol{\%=60\%}$)

|  | ApA/TpT | ApC/GpT | ApG/CpT | ApT | CpA/TpG | CpC/GpG | CpG | GpA/TpC | GpC | TpA | GC% |
| --- | --- | --- | --- | --- | --- | --- | --- | --- | --- | --- | --- |
| Proportion_exp_ vs. Proportion_ini_ | ↑ | ↓ | ↑ | ↑ | ↑ | ↓ | ↓ | ↓ | ↓ | ↔ | ↓ |
| Proportion_obs_ vs*.* Proportion_ini_ | ↑ | ↓ | ↑↓ | ↑ | ↑ | ↓ | ↓ | ↑↓ | ↓ | ↑ | ↓ |
| *Bos Taurus* (cattle) | 9.31% | 11.62% | 13.35% | 5.46% | 17.20% | 16.65% | 3.80% | 10.69% | 7.92% | 4.00% | 54.80% |
| *Canis lupus familiaris* (dog) | 9.22% | 11.73% | 13.47% | 5.37% | 17.16% | 16.53% | 3.84% | 10.78% | 7.90% | 4.00% | 54.84% |
| *Gallus gallus* (chicken) | 9.10% | 11.37% | 13.17% | 5.74% | 17.11% | 16.83% | 3.89% | 10.90% | 7.89% | 4.00% | 54.89% |
| *Pan troglodytes* (chimpanzee) | 9.23% | 11.79% | 13.40% | 5.41% | 17.24% | 16.60% | 3.76% | 10.77% | 7.80% | 4.00% | 54.76% |
| *Danio rerio* (zebrafish) | 9.11% | 11.84% | 12.93% | 5.29% | 16.46% | 17.07% | 4.54% | 10.89% | 7.87% | 4.00% | 55.54% |
| *Homo sapiens* (human) | 9.17% | 11.94% | 13.49% | 5.34% | 17.27% | 16.51% | 3.73% | 10.83% | 7.72% | 4.00% | 54.98% |
| *Mus musculus* (house mouse) | 9.34% | 11.57% | 13.41% | 5.54% | 17.40% | 16.59% | 3.60% | 10.66% | 7.89% | 4.00% | 54.60% |
| *Papio anubis* (olive baboon) | 9.16% | 11.91% | 13.48% | 5.33% | 17.20% | 16.52% | 3.80% | 10.84% | 7.76% | 4.00% | 54.80% |
| *Ovis aries* (sheep) | 9.28% | 11.67% | 13.34% | 5.44% | 17.17% | 16.66% | 3.83% | 10.72% | 7.89% | 4.00% | 54.83% |
| *Sus scrofa* (pig) | 9.18% | 11.73% | 13.42% | 5.34% | 17.01% | 16.58% | 3.99% | 10.82% | 7.93% | 4.00% | 54.99% |

*Note: The values presented were obtained by application of MDM to the assumed initial genome state with GC_ini_% = 60%; the symbols‘↑’, ‘↓’ and ‘*↔*’represent an increase, decrease, and no-change of the dinucleotide proportions expected/calculated (Proportion_exp_) or observed (Proportion_obs_；see Table 1) in genomes relative to the assumed initial proportions (Proportion_ini_; see Supplementary Table 6), respectively.*

Supplementary Table 3 Proportions/frequencies of the 16 dinucleotides in the assumed initial state of genomes with $\mathbf{GC}_{\mathbf{ini}}\boldsymbol{\%=}$50%

|  | ApA/TpT | ApC/GpT | ApG/CpT | ApT | CpA/TpG | CpC/GpG | CpG | GpA/TpC | GpC | TpA |
| --- | --- | --- | --- | --- | --- | --- | --- | --- | --- | --- |
| *Bos Taurus* (cattle) | 12.5% | 12.5% | 12.5% | 6.25% | 12.5% | 12.5% | 6.25% | 12.5% | 6.25% | 6.25% |
| *Canis lupus familiaris* (dog) | 12.5% | 12.5% | 12.5% | 6.25% | 12.5% | 12.5% | 6.25% | 12.5% | 6.25% | 6.25% |
| *Gallus gallus* (chicken) | 12.5% | 12.5% | 12.5% | 6.25% | 12.5% | 12.5% | 6.25% | 12.5% | 6.25% | 6.25% |
| *Pan troglodytes* (chimpanzee) | 12.5% | 12.5% | 12.5% | 6.25% | 12.5% | 12.5% | 6.25% | 12.5% | 6.25% | 6.25% |
| *Danio rerio* (zebrafish) | 12.5% | 12.5% | 12.5% | 6.25% | 12.5% | 12.5% | 6.25% | 12.5% | 6.25% | 6.25% |
| *Homo sapiens* (human) | 12.5% | 12.5% | 12.5% | 6.25% | 12.5% | 12.5% | 6.25% | 12.5% | 6.25% | 6.25% |
| *Mus musculus* (house mouse) | 12.5% | 12.5% | 12.5% | 6.25% | 12.5% | 12.5% | 6.25% | 12.5% | 6.25% | 6.25% |
| *Papio anubis* (olive baboon) | 12.5% | 12.5% | 12.5% | 6.25% | 12.5% | 12.5% | 6.25% | 12.5% | 6.25% | 6.25% |
| *Ovis aries* (sheep) | 12.5% | 12.5% | 12.5% | 6.25% | 12.5% | 12.5% | 6.25% | 12.5% | 6.25% | 6.25% |
| *Sus scrofa* (pig) | 12.5% | 12.5% | 12.5% | 6.25% | 12.5% | 12.5% | 6.25% | 12.5% | 6.25% | 6.25% |

*Note: GC_ini_% represents the assumed initial GC content.*

Supplementary Table 4 Information of the 10 vertebrate genomes

| Species | Accession number | Number of autosomes | Total size (Mb) | Genome length | Name | Submitter |
| --- | --- | --- | --- | --- | --- | --- |
| *Bos Taurus* (cattle) | NC_007299.6~ NC_007330.6 | 29 | 2522.20 | 2515634028 | *Bos_taurus_UMD_3.1.1* | Center for Bioinformatics and Computational Biology, University of Maryland |
| *Canis lupus familiaris* (dog) | NC_006583.3~ NC_006620.3 | 38 | 2203.76 | 2194412731 | *Canis lupus familiaris CanFam3.1* | Dog Genome Sequencing Consortium |
| *Gallus gallus* (chicken) | NC_006088.4~ NC_006111.4  NC_006112.3,  NC_006113.4~ NC_006115.4  NC_028739.1, NC_028740.1  NC_006119.3, NC_008465.3 | 32 | 933.07 | 922974567 | *GCA_000002315.4 Gallus_gallus-5.0* | International Chicken Genome Consortium |
| *Pan troglodytes* (chimpanzee) | NC_006468.4~ NC_006489.4 | 23 | 2785.21 | 2695010692 | *Pan troglodytes Pan_tro 3.0* | The International Chimpanzee Chromosome 22 Consortium |
| *Danio rerio* (zebrafish) | NC_007112.7~ NC_007136.7 | 25 | 1345.07 | 1338589074 | *Danio rerio GRCz11* | Genome Reference Consortium |
| *Homo sapiens* (human) | NC_000001.11~ NC_000022.11 | 22 | 2875.03 | 2684551162 | *GCA_000001405.26 GRCh38.p11* | Genome Reference Consortium |
| *Mus musculus* (house mouse) | NC_000067.6, NC_000068.7  NC_000069.6~ NC_000085.6 | 19 | 2462.75 | 2395908738 | *Mus musculus GRCm38.p6* | Genome Reference Consortium |
| *Papio anubis* (olive baboon) | NC_018152.1~ NC_018171.1 | 20 | 2581.20 | 2541422570 | *GCF_000264685.2 Panu_2.0* | Baylor College of Medicine |
| *Ovis aries* (sheep) | NC_019458.2~ NC_019483.2 | 26 | 2449.65 | 2427812878 | *Ovis aries Oar_v4.0* | International Sheep Genome Consortium |
| *Sus scrofa* (pig) | NC_010443.5, NC_010444.4  NC_010445.4, NC_010446.5  NC_010447.5, NC_010448.4  NC_010449.5, NC_010450.4  NC_010451.4, NC_010452.4  NC_010453.5, NC_010454.4  NC_010455.5, NC_010456.5  NC_010457.5, NC_010458.4  NC_010459.5, NC_010460.4 | 18 | 2265.78 | 2264066289 | *Sus scrofa Sscrofa11.1* | The Swine Genome Sequencing Consortium (SGSC) |

*Note: The genome length is the number of nucleotides counted merely on the autosomes with the removal of gaps.*

Supplementary Table 5 Proportions/frequencies of the 16 dinucleotides in the assumed initial state of genomes with $\mathbf{GC}_{\mathbf{ini}}\boldsymbol{\%=}$40%

|  | ApA/TpT | ApC/GpT | ApG/CpT | ApT | CpA/TpG | CpC/GpG | CpG | GpA/TpC | GpC | TpA |
| --- | --- | --- | --- | --- | --- | --- | --- | --- | --- | --- |
| *Bos Taurus* (cattle) | 18% | 12% | 12% | 9% | 12% | 8% | 4% | 12% | 4% | 9% |
| *Canis lupus familiaris* (dog) | 18% | 12% | 12% | 9% | 12% | 8% | 4% | 12% | 4% | 9% |
| *Gallus gallus* (chicken) | 18% | 12% | 12% | 9% | 12% | 8% | 4% | 12% | 4% | 9% |
| *Pan troglodytes* (chimpanzee) | 18% | 12% | 12% | 9% | 12% | 8% | 4% | 12% | 4% | 9% |
| *Danio rerio* (zebrafish) | 18% | 12% | 12% | 9% | 12% | 8% | 4% | 12% | 4% | 9% |
| *Homo sapiens* (human) | 18% | 12% | 12% | 9% | 12% | 8% | 4% | 12% | 4% | 9% |
| *Mus musculus* (house mouse) | 18% | 12% | 12% | 9% | 12% | 8% | 4% | 12% | 4% | 9% |
| *Papio anubis* (olive baboon) | 18% | 12% | 12% | 9% | 12% | 8% | 4% | 12% | 4% | 9% |
| *Ovis aries* (sheep) | 18% | 12% | 12% | 9% | 12% | 8% | 4% | 12% | 4% | 9% |
| *Sus scrofa* (pig) | 18% | 12% | 12% | 9% | 12% | 8% | 4% | 12% | 4% | 9% |

*Note: GC_ini_% represents the assumed initial GC content.*

Supplementary Table 6 Proportions/frequencies of the 16 dinucleotides in the assumed initial state of genomes with $\mathbf{GC}_{\mathbf{ini}}\boldsymbol{\%=}$60%

|  | ApA/TpT | ApC/GpT | ApG/CpT | ApT | CpA/TpG | CpC/GpG | CpG | GpA/TpC | GpC | TpA |
| --- | --- | --- | --- | --- | --- | --- | --- | --- | --- | --- |
| *Bos Taurus* (cattle) | 8% | 12% | 12% | 4% | 12% | 18% | 9% | 12% | 9% | 4% |
| *Canis lupus familiaris* (dog) | 8% | 12% | 12% | 4% | 12% | 18% | 9% | 12% | 9% | 4% |
| *Gallus gallus* (chicken) | 8% | 12% | 12% | 4% | 12% | 18% | 9% | 12% | 9% | 4% |
| *Pan troglodytes* (chimpanzee) | 8% | 12% | 12% | 4% | 12% | 18% | 9% | 12% | 9% | 4% |
| *Danio rerio* (zebrafish) | 8% | 12% | 12% | 4% | 12% | 18% | 9% | 12% | 9% | 4% |
| *Homo sapiens* (human) | 8% | 12% | 12% | 4% | 12% | 18% | 9% | 12% | 9% | 4% |
| *Mus musculus* (house mouse) | 8% | 12% | 12% | 4% | 12% | 18% | 9% | 12% | 9% | 4% |
| *Papio anubis* (olive baboon) | 8% | 12% | 12% | 4% | 12% | 18% | 9% | 12% | 9% | 4% |
| *Ovis aries* (sheep) | 8% | 12% | 12% | 4% | 12% | 18% | 9% | 12% | 9% | 4% |
| *Sus scrofa* (pig) | 8% | 12% | 12% | 4% | 12% | 18% | 9% | 12% | 9% | 4% |

*Note: GC_ini_% represents the assumed initial GC content.*

Supplementary Table 7 Numbers of the trinucleotides NpCpG and CpGpM in the 10 vertebrate genomes

|  | S_ACG_ | S_CCG_ | S_GCG_ | S_TCG_ | S_CGA_ | S_CGC_ | S_CGG_ | S_CGT_ |
| --- | --- | --- | --- | --- | --- | --- | --- | --- |
| *Bos Taurus* (cattle) | 6644375 | 6137060 | 4916691 | 5997553 | 5982518 | 4909510 | 6133470 | 6670181 |
| *Canis lupus familiaris* (dog) | 5254229 | 5657268 | 4212111 | 4674584 | 4671666 | 4210351 | 5655013 | 5261162 |
| *Gallus gallus* (chicken) | 2814832 | 1892253 | 1790917 | 1787847 | 1787286 | 1794901 | 1890155 | 2813507 |
| *Pan troglodytes* (chimpanzee) | 6532056 | 6523676 | 5563353 | 5728861 | 5718577 | 5566623 | 6513422 | 6549324 |
| *Danio rerio* (zebrafish) | 6239560 | 4522114 | 5453402 | 5396818 | 5397763 | 5461310 | 4530850 | 6221971 |
| *Homo sapiens* (human) | 6011035 | 6193556 | 5251724 | 5343371 | 5336719 | 5250531 | 6188814 | 6023622 |
| *Mus musculus* (house mouse) | 5443950 | 4967044 | 3914464 | 4737550 | 4733280 | 3912152 | 4966359 | 5451217 |
| *Papio anubis* (olive baboon) | 6563005 | 7294242 | 6129304 | 5724016 | 5717490 | 6133265 | 7293465 | 6566347 |
| *Ovis aries* (sheep) | 6556797 | 6132085 | 5073909 | 5837952 | 5820124 | 5070330 | 6125256 | 6585033 |
| *Sus scrofa* (pig) | 6535092 | 6963077 | 5244237 | 5794240 | 5781468 | 5239302 | 6969102 | 6546774 |

*Note: Only the autosomes of each genome were included in the statistical analyses.*


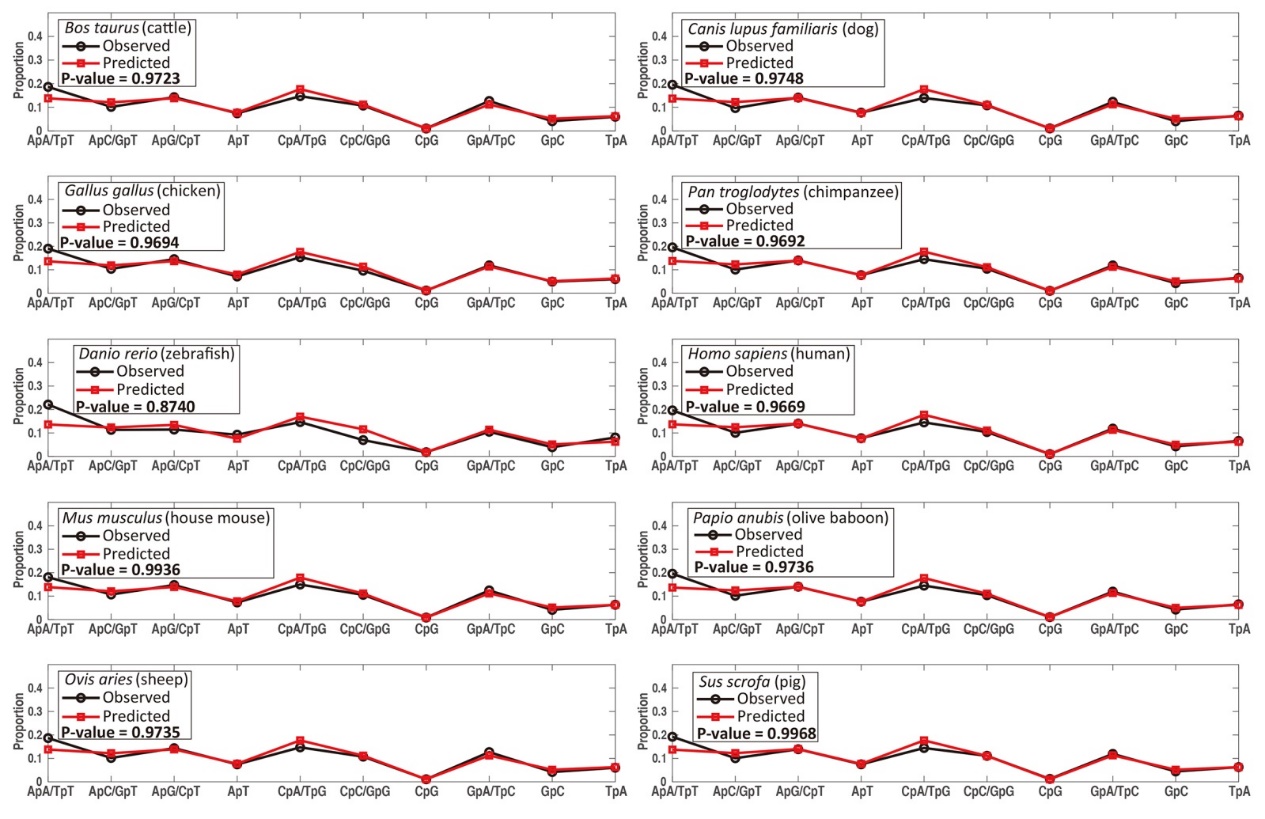
**Supplementary Fig. 1** Comparison between the observed and expected frequencies/proportions of the 16 dinucleotides. Note that the expected frequencies were obtained using GC_ini_% = 50%. P-value shown in the inserted box was obtained by performing the paired t-test on the observed and expected frequencies of the 16 dinucleotides for each genome.
